# The Fractal Patterns of Words in a Text: A Method for Automatic Keyword Extraction

**DOI:** 10.1371/journal.pone.0130617

**Published:** 2015-06-19

**Authors:** Elham Najafi, Amir H. Darooneh

**Affiliations:** Department of Physics, University of Zanjan, Zanjan, Iran; University of Jaén, SPAIN

## Abstract

A text can be considered as a one dimensional array of words. The locations of each word type in this array form a fractal pattern with certain fractal dimension. We observe that important words responsible for conveying the meaning of a text have dimensions considerably different from one, while the fractal dimensions of unimportant words are close to one. We introduce an index quantifying the importance of the words in a given text using their fractal dimensions and then ranking them according to their importance. This index measures the difference between the fractal pattern of a word in the original text relative to a shuffled version. Because the shuffled text is meaningless (i.e., words have no importance), the difference between the original and shuffled text can be used to ascertain degree of fractality. The degree of fractality may be used for automatic keyword detection. Words with the degree of fractality higher than a threshold value are assumed to be the retrieved keywords of the text. We measure the efficiency of our method for keywords extraction, making a comparison between our proposed method and two other well-known methods of automatic keyword extraction.

## Introduction

Language is the human capability for communication via vocal or visual signs. Language can be regarded as a complex system [1], where words are constituents which interact with each other to form particular patterns. Such patterns represent human thoughts, feelings, will, and knowledge which are called meaning. Human language is unique among other communication systems, because there are a lots of words to express the immaterial and intellectual concepts. In addition, the existence of synonymy, polysemy and so on increases its complexity. Texts, as the written form of language, inherit its complexity. A text can be partially understood through regularities in spatial distribution of words and their frequencies. Research has shown that regularity in a text can be expressed as a power law relationship. One of the most well-known power laws is Zipf’s law, which shows that if we rank the words in a text from the most common to the least, the frequency of each word is inversely proportional to its rank [2]. A related law, Heaps’ law, shows another universal feature of texts: the number of distinct words in a text (i.e., number of word *types*), changes with the text size (i.e., the number of *tokens*) in the form of a power law [3]. Another level of regularity is evident only through the pattern of words throughout a text. A text is not just a random collection of words; we can only call this collection a text if it has meaning. In other words, the words in a text must be placed in a specific order to impart meaning. Many power laws cannot capture this fact: any random shuffling process drastically destroys the meaning of a text, but Zipf’s law remains unchanged and Heaps’ law changes only very slightly [4].

The particular arrangement of words in a specific order arises for two reasons. First, grammatical rules determine where words should be placed within a sentence and specify the position of verbs, nouns, adverbs, and other parts of speech. Grammatical rules make short range correlations between the sequences of words in a sentence. Secondly, a text derives meaning from how the words are arranged throughout. This ordering is called semantic ordering, and acts across the whole range of the text, hence the long-range correlation can be seen between the positions of any word. The broad meaning of a text also means that different word types have different importance in a text. We can distinguish between two kinds of content words in a text: those which are related to the subject of the text (i.e., the important words), and all others that are irrelevant to it. For a text in cosmology, words like *universe, space, big-bang*, and *inflation* are important words. Other words such as *is, fact, happening, etc*., are irrelevant to the topic of the text. Finding an index for quantifying the importance of words in a given text is crucial to detecting keywords automatically, and provides a very useful starting point for text summarization, document categorization, machine translation and other matters related to automatic information retrieval. Automating these processes is of increasing importance given the increasing size of available information yet limited man-power.

In the current paper, we use the concept of fractal to assign an importance value to every word in a given text. A fractal is a mathematical object (e.g., a set of points in Euclidean space) that has repeating patterns at every scales, it means at any magnification there is a smaller piece of the object that is similar to the whole; this property is called self-similarity. The fractal dimension shows how detail of a fractal pattern changes with scale. It is used as an index of complexity. The fractal dimension of a set is equal or less than the topological dimension of space that the set is embedded in it. We claim that the positions of a word type within the text array form a fractal pattern with a specified dimension that is a positive value less than or equal to one. Based on this fact, an index is presented for ranking the vocabulary words of a given text. The difference between the pattern of a word in the original text versus a randomly shuffled version shows its importance: words with a greater differential between the original and shuffled texts are more important. We compare this approach with other more well-known methods of keyword extraction.

In the following section we review previous research reporting a kind of fractal structure in texts, in order to show that our method is novel. Then we review some basic ideas for keyword extraction which are useful for understanding the different principles currently at work in the field. Finally, we describe our method and how it could be evaluated, and report the results for a sample book.

## Background and Related works

### Fractal Structures in Texts

In 1980 G. Altmann made a formula for quantifying of the Menzerath’s law [5]. Menzerath-Altmann law says there is a relation between size of a construct and size of its constituents. A system like a language has different levels or constructs, such as syllables, words, syntactic constructions, clauses, sentences and semantic constructs. According to Menzerath-Altmann law, when the size of a construct increases, the size of its constituents decreases, and this holds at every level. Thus, a certain kind of self-similarity exists for each level [6, 7]. Fractal dimension can be calculated for each level. The fractal dimension of a given text is the average value of fractal dimension of levels [8].

For quantitative calculations, texts are usually mapped into time series. A text can be considered as a one dimensional array where elements can be either characters, words or sentences. Ausloos built two time series by replacing each word in the text by their length or frequency [9, 10]. He quantified the complexity in a written text by examining the fractal pattern of its corresponding length and frequency time series, discovering that resulting fractal patterns may be used as an authorship indicator. Furthermore, these length and frequency time series also gave indications of the semantic complexity of the text.

Eftekhari worked on letters instead of words as the constituents of a text, finding that if letter types in a text are ranked from the most common to the least, the frequency of each letter type would be inversely proportional to its rank [11] (i.e., simillar to Zipf’s law). If frequency of letter types is plotted versus their ranks in a double logarithmic scale, a straight line is obtained. He called the slope of this line Zipf’s dimension. He also suggested a method for calculating fractal dimension of texts, declaring that if letter types are ranked in alphabetical order and frequency of letter types is plotted against their ranks, the slope of such a diagram would be fractal dimension of the literature. Nevertheless, since the data which is used is too disperse he used the so-defined fractal dimension. He also showed that texts exhibit changes in fractal dimension similar to corresponding Zipf’s dimension which vary according to the text’s size.

### Principles for Keyword Extraction

The first method based on Zipf’s analysis of word frequency for keyword extraction was proposed by Luhn [12]. He plotted the Zipf diagram of words, then eliminated words with high and low frequencies, and declared that the words remaining in the mid-range frequencies are the most important words of a text. There are some problems with this method; it omits some important words which have very low frequencies, and may also mistakenly take some common words with mid-range frequencies as keywords. To overcome this deficiency, Ortuño *et al*. proposed a method based on the concept that important words form clusters [13]. They used standard deviation of distance between consecutive occurrences of a particular word as a measure of word clustering. Words with large standard deviations tend to form clusters and so are more important. Carpena *et al*. improved this method and introduced the *C Value* for measuring the importance of words [14] based on their clustering distributions (we review this method in the appendix section in contrast to our own). Another method based on clustering was proposed by Zhou and Slater [15]. They used the density fluctuations of words as a measure of clustering. The method was useful to reduce significance of common words. Mihalcea and Tarau used a method based on the graph theory for detecting the keywords [16]. The text is regarded as a graph with word types nodes with edges occuring between two words where they are adjacent in the text. To extract keywords they introduced the concept of *TextRank*, calculated similarly to PageRank which is used in the Google search engine for ranking the web pages. TextRank works by counting the number and weight of links to a node to determine importance of the node. The more important nodes are likely to receive more links from other nodes. Words with higher values of TextRank are more important. Herrera and Pury suggested an entropic method for word ranking based on the relative frequency of words in each part of the text [17] (this method is also reviewed in the appendix in contrast to our own). Mehri and Darooneh used several entropic metrics to extract keywords [18]. In particular, they found that cumulative distribution of distances between consecutive occurrences of a word type follows:

$$\begin{array}{c} P={\left[1+(q-1)\beta x\right]}^{\frac{1}{1-q}} \end{array}$$ (1)

where x is distance between consecutive occurrences of a word type, *β* is a constant, and *q* is a positive value. They ranked words according to *q* value. The value of *q* in the case of important words is larger than the case of common words [19].

## Methods

### The Degree of Fractality

Text is a certain arrangement of words in one dimensional array that carries a meaning. Any random shuffling of the words across the text significantly reduces its meaning, hence the ordering of the words is important for representation of the meaning. In other words, the meaning shows a kind of regularity in a text. This regularity also manifests itself in pattern of occurrences of each word in the text array. If we consider the text array as a one dimensional space, the spatial pattern of occurrences of any vocabulary word will form a fractal set or simply a fractal. We can assign a fractal dimension to any word in a given text using the practical method of Box Counting. Using this method, the fractal dimension of a word is generally between 0 and 1.

In Box-Counting the space is divided into boxes. Each box that contains a component of the fractal set is called a filled box. The fractal law is a power law relationship between the number of filled boxes and the box-size [20].

To calculate the fractal dimension of a word by box-counting method, the text array is divided into boxes of size *s*, we place each *s* consecutive words in a box. The number of such boxes is *N*_*s*_ = *N*/*s* where *N* is the length of the text. If the considered word appears in one of the boxes, that box is a filled box, *N*_*b*_(*s*) stands for the number of filled boxes. A power law relationship exists between the number of filled boxes and the box size *s* as follows,

$$\begin{array}{c} N_{b}\left(s\right)\propto s^{-D} \end{array}$$ (2)

*D* is the fractal dimension of the word. Fractal dimension is obtained by measuring the slope of log-log plot of *N*_*b*_(*s*) versus *s*. It is worth noting that here the box size is an integer number, and in practice, we expect to see the power law behavior for the large box sizes.

As we noted earlier, the fractal dimension for any word is between 0 and 1. When all occurrences of a word are distributed uniformly across the text, all of the boxes have the same probability of containing a token of the word. Therefore, in this particular case, the number of filled boxes has the maximum possible value. In other cases, some of the boxes may contain more than one occurrence; this results in some of the other boxes remaining empty, and the number of filled boxes is less than this limiting value.

In a shuffled text, all of the words are distributed uniformly. For small scales, when the number of boxes is greater than the frequency of a word type, the number of filled boxes is expected to be approximately equal to the frequency of the word type. By increasing the box size, the number of filled boxes will be decreased. In large scales, the fact that the number of filled boxes is maximum makes the slope of the log-log plot of *N*_*b*_(*s*) versus *s* close to one; the upper limit for slope. The following equation indicates our conjecture on the number of filled boxes for a word in the shuffled text against the box size, consistent with the above facts.

$$\begin{array}{c} N_{b}^{sh.}\left(s,\omega \right)=\frac{M}{1+\left(\frac{M-1}{N-1}\right)\left(s-1\right)} \end{array}$$ (3)

where *M* is frequency of the word *ω*.

The fractal dimension is the slope of the line of best fit on the log-log plot of the number of filled boxes against the box size. In practice, the choice of the fitting range is very important and definitely has influence on the value of the fractal dimension. Unfortunately, there is no way to automatically choose the most appropriate fitting range. Instead of the fractal dimension, we propose an index which is used to quantify the fractality of the word pattern in another way. The degree of fractality is defined as,

$$\begin{array}{c} d_{f}\left(\omega \right)=\sum_{s}\log\left(\frac{N_{b}^{sh.}\left(s,\omega \right)}{N_{b}\left(s,\omega \right)}\right) \end{array}$$ (4)

where *ω* is a particular word. The degree of fractality, *d*_*f*_, measures the difference between the pattern of occurrences of a word in the original and shuffled text. We use the logarithm in the definition of this index to avoid domination of the values for small box sizes. The degree of fractality is a suitable quantity for ranking the words of a text. In computing the degree of fractality, we only need to find the number of filled boxes for any scale. Unlike the process of computation of the fractal dimension, data regression is not required. Moreover, we are not faced with the problem of determining the fitting range for each word. The larger value for the degree of fractality means the distribution pattern of a word has more differences with the uniform distribution.

### Evaluation of the Method

The degree of fractality gives an importance value for every word type in a given text. Using this value, we are able to list the words from greatest to least importance. The top-ranked words of the list are assumed as keywords.

A comparison with a manually created list of keywords allows for an approximate evaluation of the efficiency of our method. It is important to know how the list of the relevant keywords is prepared for a given book. In our experience we assume that the manually created glossary of a book is a good candidate for providing the relevant keywords of the book. The glossary of a book should be prepared by author or some experts of the field thus it is reliable to be selected as our reference data.

The following two issues are important when we have comparison between the list of relevant and retrieved keywords. First, it is important to compute how many words are common in the two lists if both of them have the same size. Second, what fraction of the retrieved list should be selected to include all the relevant keywords? In binary classification analysis, recall and precision are two metrics which consider the above issues respectively. The recall and precision are calculated as follows according to Herrera and Pury’s suggestion [17]. These are well-known metrics for evaluation of keyword extraction methods.

$$\begin{array}{c} R=\frac{N_{c}}{N_{gloss}} \end{array}$$ (5)

$$\begin{array}{c} P=\frac{N_{gloss}}{N_{last}} \end{array}$$ (6)

Where *N*_*gloss*_ is the size of list of relevant keywords (glossary), *N*_*c*_ is the number of common keywords in two lists, which have the rank less than *N*_*gloss*_ and *N*_*last*_ stands for the last position of relevant keywords in the list of retrieved words. It is worth noting again that these metrics cannot precisely determine the accuracy of the keyword detection methods. According to our experience, they depend on the data processed (selected book, its genre) and on how the list of relevant keywords is prepared.

There is another method for calculating recall and precision that is suggested by Mehri and Darooneh [18]. In this method words with degree of fractality higher than a threshold value are selected as retrieved keywords. The threshold value is choosen such that some percentage of ranked list of words is selected as the retrieved keywords in each step. Then, number of keywords which is the same between glossary and this new list is counted. Recall and precision are calculated as follows.

$$\begin{array}{c} R=\frac{N_{c}}{N_{gloss}} \end{array}$$ (7)

$$\begin{array}{c} P=\frac{N_{c}}{N_{ret}} \end{array}$$ (8)

Again, *N*_*gloss*_ is the size of glossary and *N*_*c*_ is the number of keywords which are the same between glossary and selected percentage of retrieved list. *N*_*ret*_ is the size of the retrieved list to the whole vocabulary size in percent.

## Results

### Universal Properties of Texts

**Fig 1:**
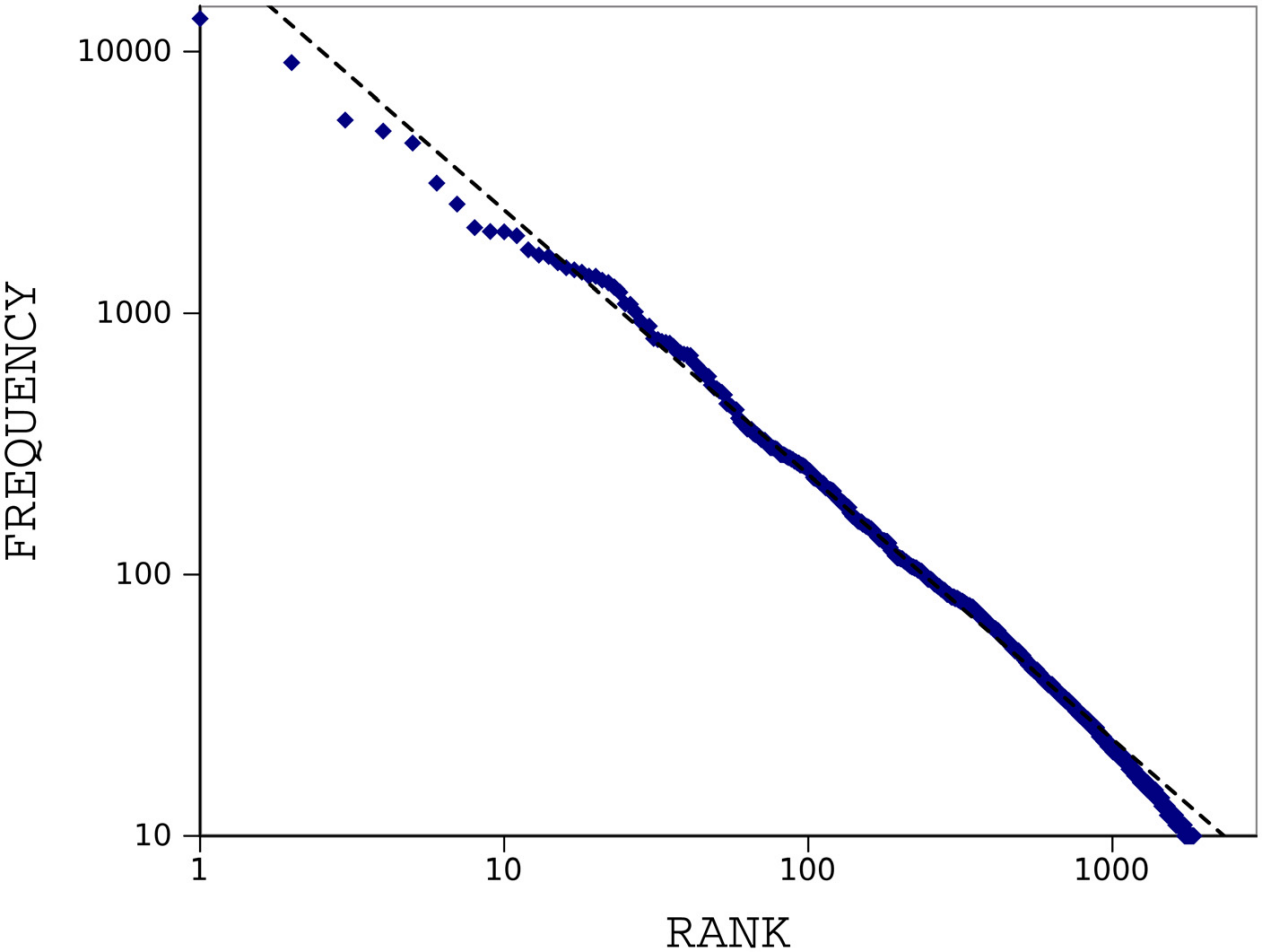
Zipf’s law for the book *The Origin of Species*. Frequency of each word is inversely proportional to its rank in form of power law. The Zipf curve follows a straight line with a slope of −1.01 when plotted on a double-logarithmic scale.

**Fig 2:**
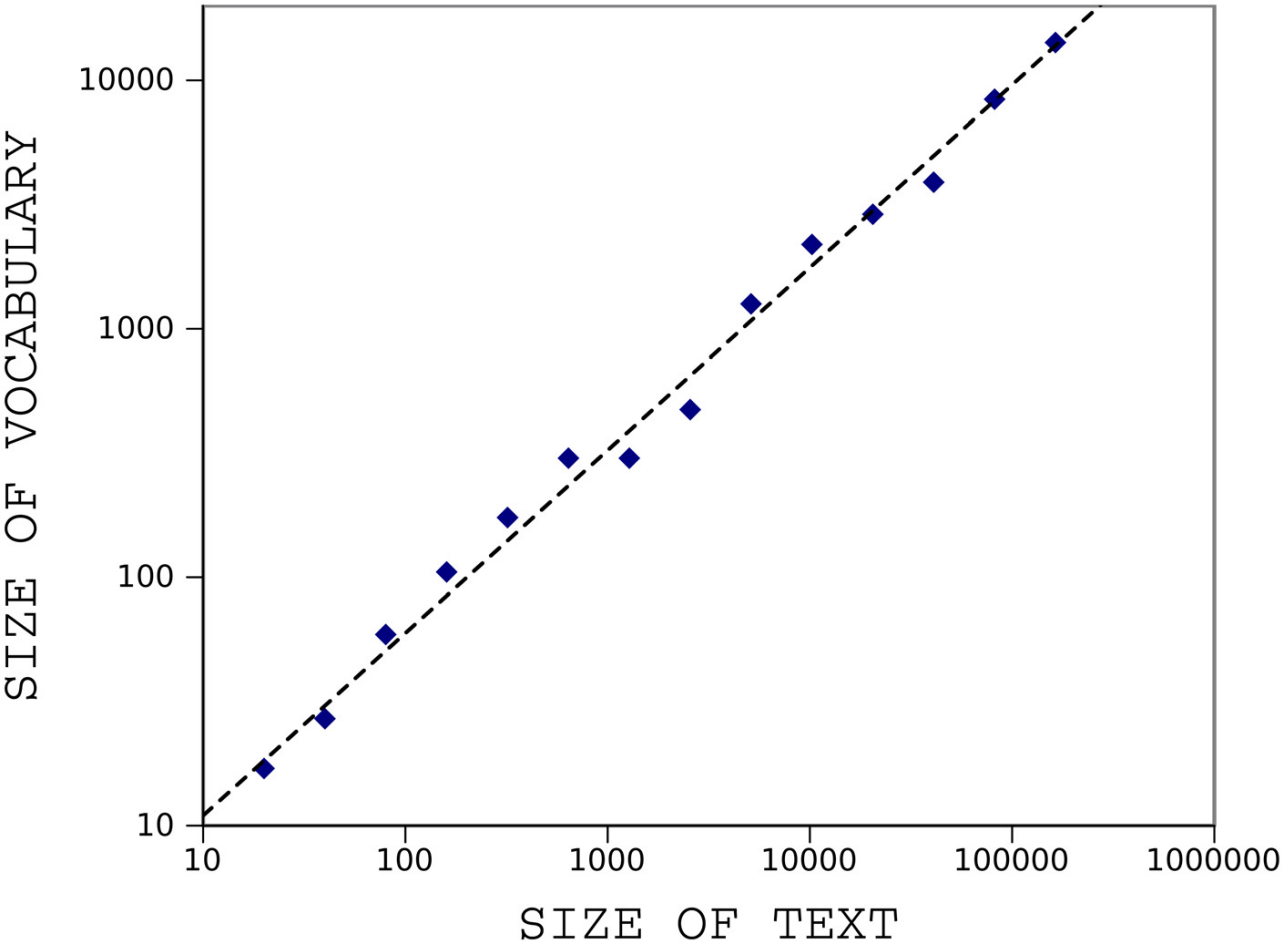
Heap’s law for the book *The Origin of Species*. Size of vocabulary increases as size of text increases, in form of power law. The Heap curve follows a straight line with a slope of 0.73 when plotted on a double-logarithmic scale.

To explain more details, we apply our method to *On The Origin of Species* by Charles Darwin [21]. The book is about evolution of populations through a process of natural selection. A digital copy of this text is freely available on *Project Gutenberg* [22]. We only keep the main body of the text and leave the others (e.g., contents, index). No other preprocessing tasks are performed except deletion of the non-alphabetic characters. The book has a total of 191740 tokens and contains 8842 distinct word types. We examined two famous regularities of texts for this book, the Zipf’s and Heaps’ law. Fig 1 shows Zipf’s law for the book; frequency of each word type is plotted against word rank on a double-logarithmic scale. A straight line is obtained with a slope of −1.01. Fig 2 shows Heap’s law for the book; size of vocabulary is plotted versus size of text on a double-logarithmic scale. A straight line is obtained with a slope of 0.73.

**Fig 3:**
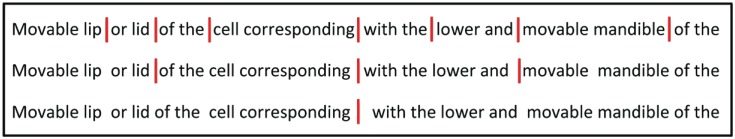
Schematic of how an instance text is devided into boxes. The number of words that is placed in a box, is the box-size. Box-Size for first row is equal to 2 and for the second and third rows are 4 and 8 respectively.

As outlined earlier the spatial distribution or pattern of ocurrences of any word in a given text exhibits self-similarity. The box counting is a practical procedure for measuring this property. In this procedure, the text is divided into boxes of size *s*, that varies from 1 to the text size. *s* = 1 means each box contains only one word, *s* = 2 means each box contains two words, and so on. A box is called filled if it contains some instances of the considered word. We chose powers of 2 for our box sizes. As an example Fig 3 illustrates division of a small part of our sample book into boxes with size 2, 4 and 8. In this example the appears in 3, 3, and 2 boxes for *s* = 2, 4, and 8 respectively.

**Fig 4:**
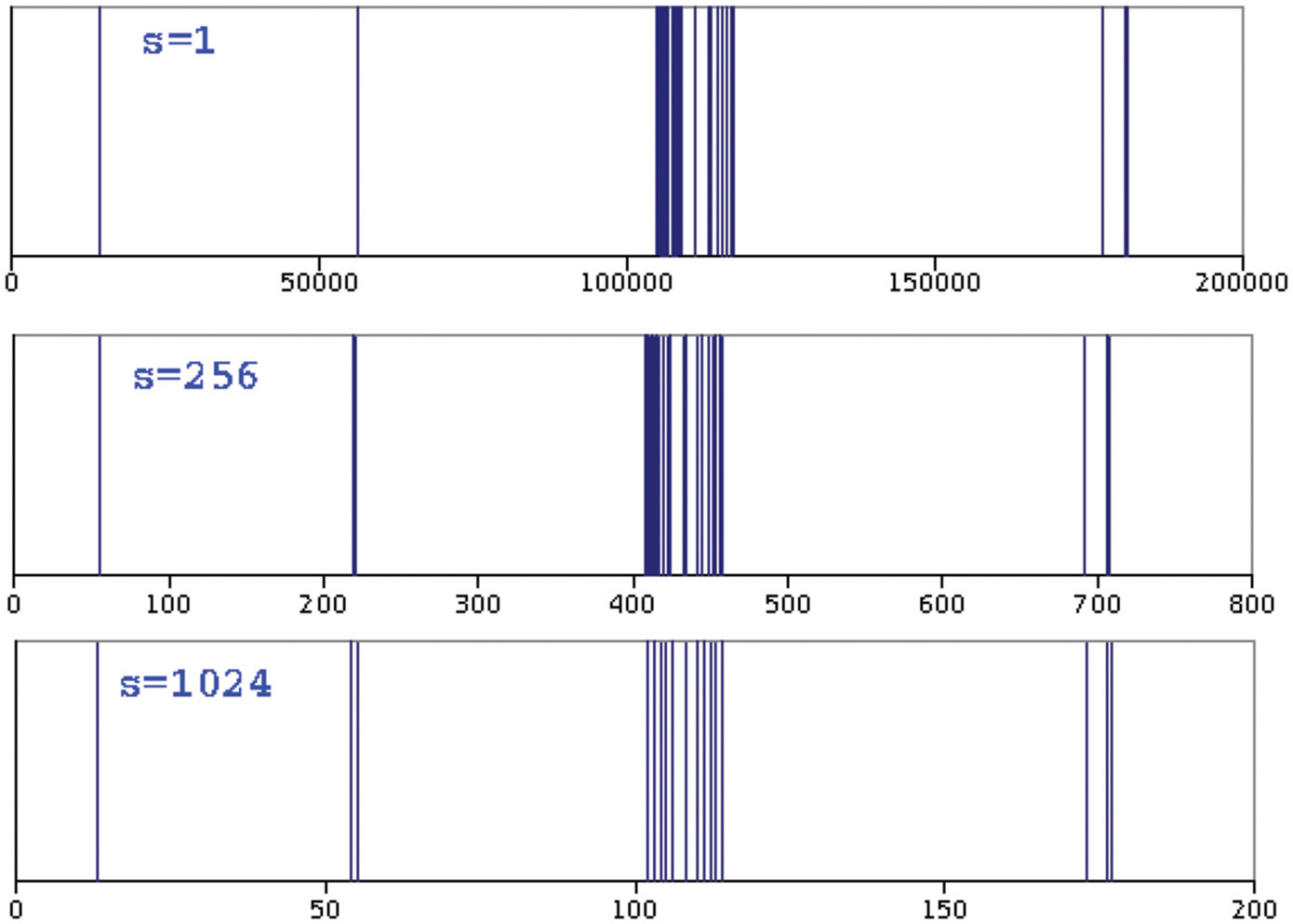
Spatial distribution of HYBRID in the book, *The Origin of Species* for three different scales. As seen, distributions in all scales, *s* = 1, *s* = 256 and *s* = 1024, are statistically the same. They have similar clusters.

Distribution of a word is self-similar if we see the same pattern for the word in all scales (in all *s*). In Fig 4 the distribution of hybrid, one of the vocabulary words in our sample book is shown in three different scales *s* = 1, *s* = 256 and *s* = 1024. As is seen in this figure, distribution of hybrid is the same in these scales.

### Ranking the words and keyword detection

**Fig 5:**
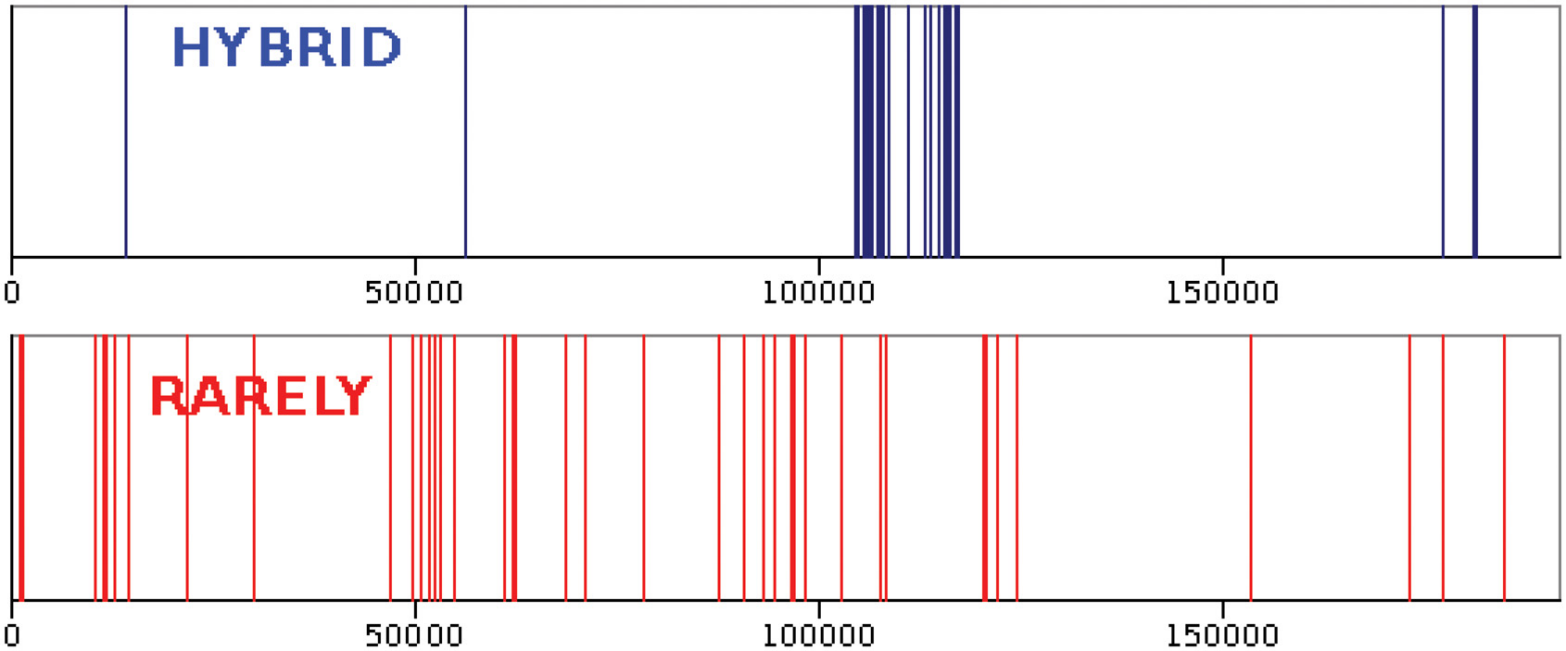
Spatial distribution of two words, HYBRID and RARELY, in the book, *The Origin of Species*. According to subject of the book, hybrid is an important and the rarely is an irrelevant word, both of them have the same frequency equal to 45. rarely is distributed in the text, uniformly but,hybrid is clustered.

All words have a self-similar pattern in the text, but with different fractal dimensions. If the word is uniformly distributed along the text its fractal dimension is close to one. For words which are clustered in text the fractal dimension is substantially less than one. Fig 5 shows distribution of two words of the instance book, hybrid and rarely. Both of them have the same frequency *M* = 45. Occurrences of hybrid form a cluster in the text while rarely has uniform distribution.

**Fig 6:**
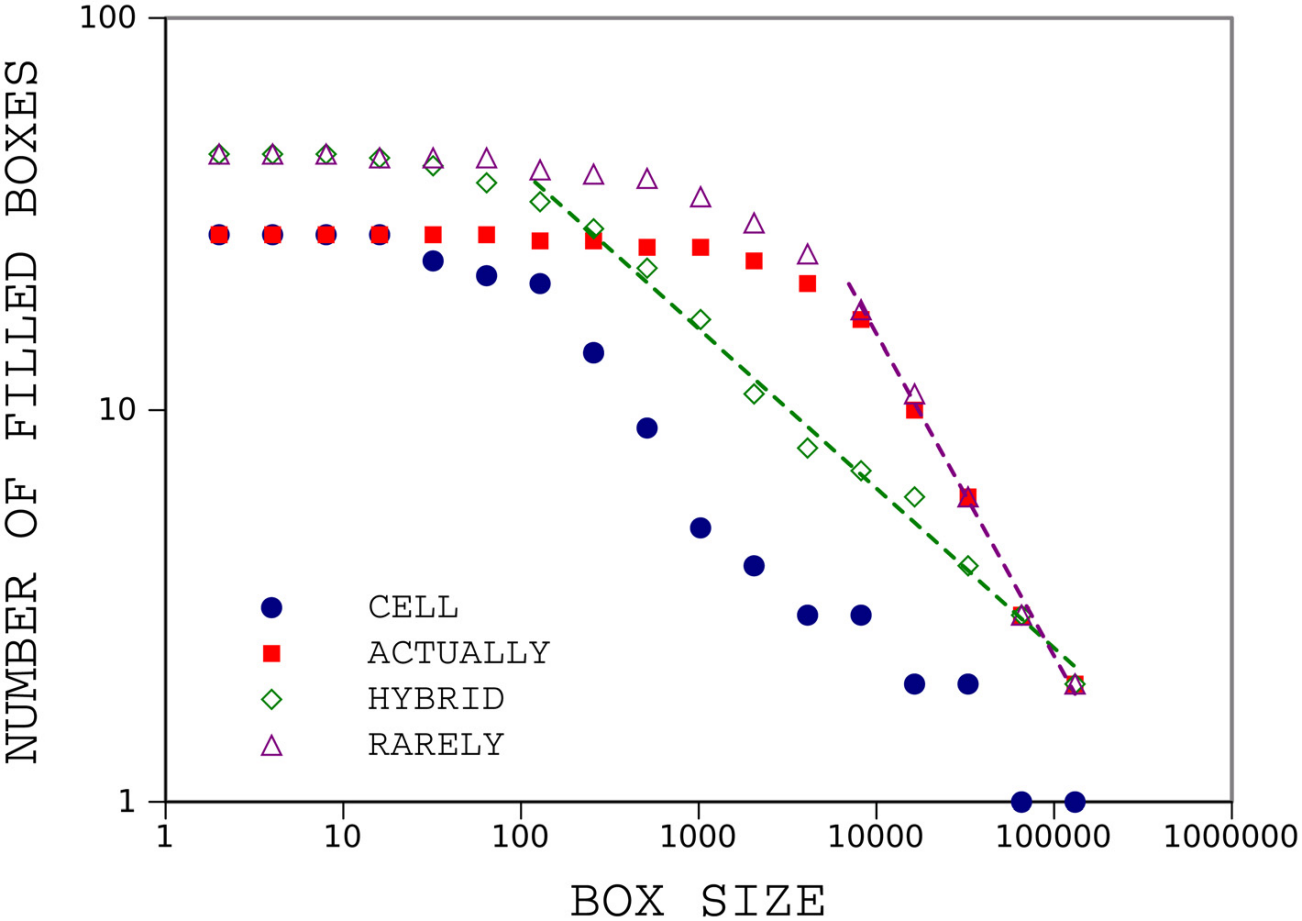
Results of box counting for, HYBRID and RARELY. The dashed line and dash dotted line demonstrate the power law regression. The fractal dimension is about 0.4 for hybrid and is close to 0.8 for rarely. The box counting result of cell and actually is also showed. The fractal dimension is about 0.4 for cell and is close to 0.8 for actually.

In Fig 6 we compute the fractal dimension for these words. hybrid has dimension 0.4 and dimension of rarely is 0.8. We also plot the results for other pair of words, cell and actually with 28 occurrences in the book for both of them. cell is clustered as same as hybrid and actually has uniform pattern like rarely.

**Fig 7:**
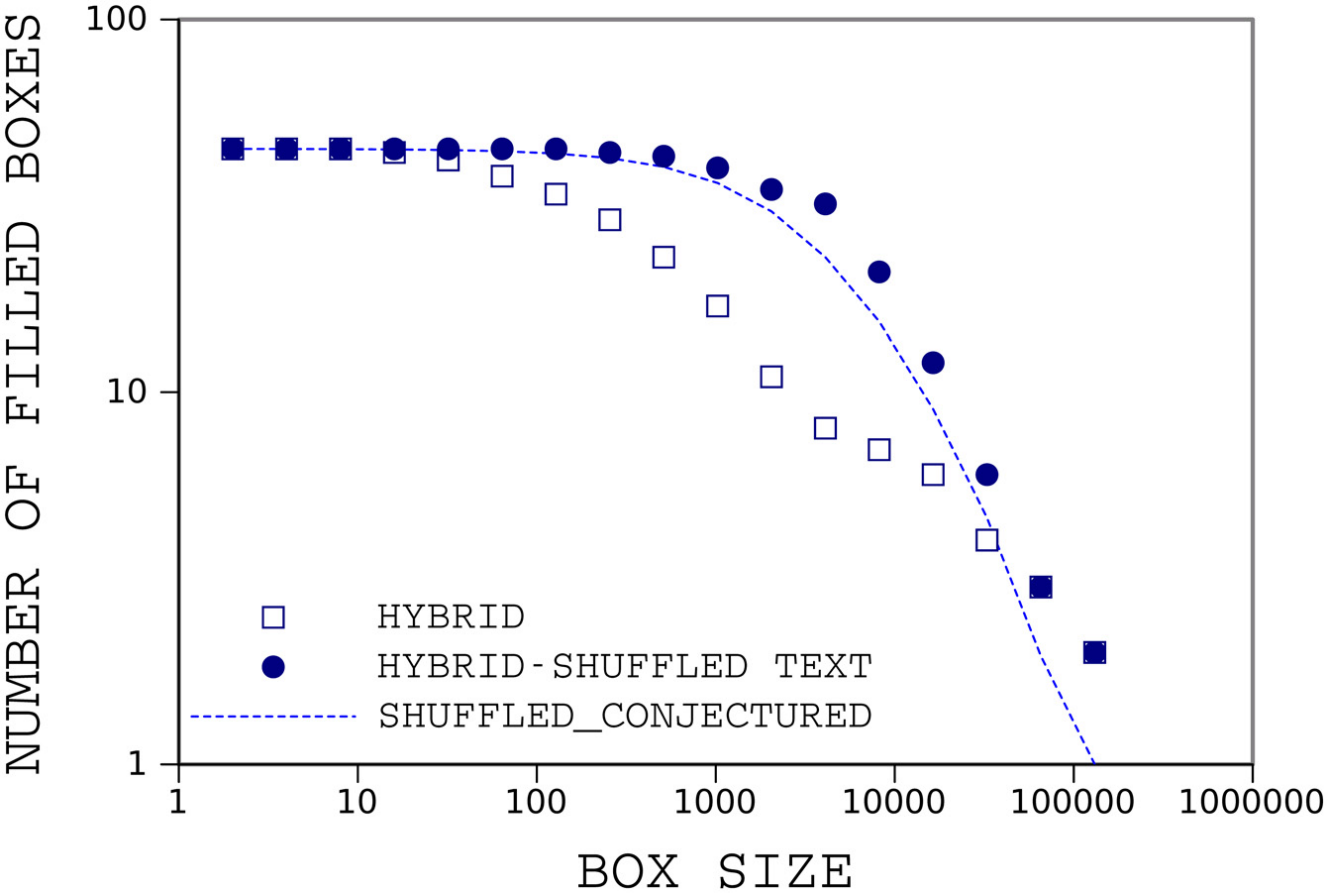
Results of box counting for distribution of HYBRID in the original and shuffled text. hybrid is an important word in the book, *The Origin of Species*. So, there is a considerable difference between box-counting of this word in the original and shuffled text.

In the shuffled text all words are distributed more uniformly and clustered words do not occur. Fig 7 illustrates the result of box counting for hybrid in our sample book and its shuffled version. Our conjecture on the number of filled boxes in the shuffled text is also plotted, showing that our conjecture has good agreement with the shuffled data.

**Fig 8:**
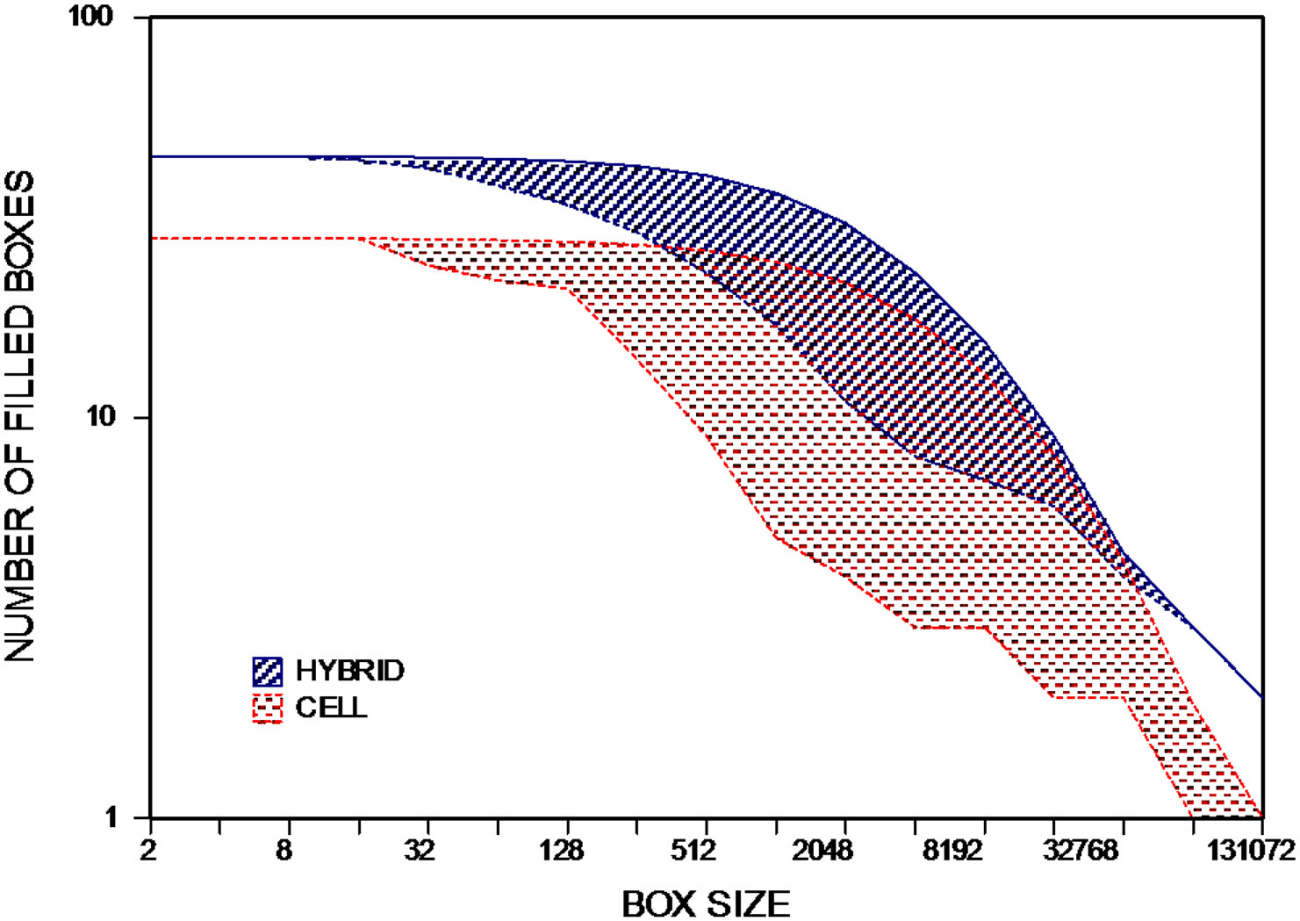
Area which is bounded between two curves for CELL and HYBRID in the box counting diagram. The curves correspond to box counting result for these two words in the original and shuffled text. The area corresponds to cell is bigger than the case of hybrid. cell is more important than hybrid in the book *The Origin of Species*.

The patterns of words that have uniform distributions change only slightly after the shuffling process, indicating that the words uniformly distributed in the original text are unimportant. The difference between patterns of a word in the original and shuffled text can be considered an indication of its importance. The degree of fractality which is defined in Eq 4 measures this difference. Fig 8 shows the degree of fractality for two words, hybrid and cell. It is clear from this figure that cell is more important than hybrid. The degree of fractality of hybrid is 8.21 and is 12.71 in the case of cell. Now we can rank all of the words according to the degree of fractality. Table 1 reports the list of twenty top-ranked words and also the first twenty frequent words for comparison. According to the subject of the book, words such as, slaves, illegitimate, saliva, and pedicellariae are important words. They also have higher degree of fractality in comparison with other words. The irrelevant words like, the, of, and, and in have lower degree of fractality, though they are very frequent in the book. It is useful to point out that function words have the lowest degree of fractality overall, but unimportant content words still have lower fractality than important keywords.

**Table 1:** List of the twenty top-ranked words according to degree of fractality (left) and the first twenty frequent words (right) from the book *The Origin of Species*. Words with high degree of fractality are important words according to subject of the book and common words have low degree of fractality. The string *un* which is placed in the second row of list of top-ranked words is a French determinant which appears four times in a single sentence. So, it is highly clustered and has high value of fractality. Because we do not perform any pre-processing to eliminate foreign words, this word appears in the list.

Words	Frequency	Fractality	Words	Frequency	Fractality
slaves	34	17.42	the	13368	2.54
un	4	16.70	of	9071	2.67
illegitimate	21	16.52	and	5482	2.79
saliva	5	16.42	in	4973	2.97
pedicellariae	15	16.03	to	4477	2.79
floated	18	15.98	a	3143	2.71
pupae	13	15.72	that	2612	2.77
wax	42	15.65	as	2122	3.16
vibracula	12	15.54	have	2051	2.79
masters	17	15.52	be	2045	2.78
avicularia	13	15.28	is	1975	2.80
dried	9	15.11	species	1745	2.42
movable	10	15.10	by	1665	2.82
segment	5	15.04	which	1646	2.76
caudicle	6	14.59	are	1556	2.69
neuters	12	14.93	or	1489	3.22
cuckoo	32	14.89	it	1462	3.04
lamellae	20	14.67	on	1432	3.12
dun	8	14.60	with	1383	3.02
bucket	7	14.59	for	1381	2.98

For small texts, word frequency becomes increasingly important. For taking into account the effect of frequency, we multiply log(*M*) by the degree of fractality, causing the most changes in degree of fractality rank in the middle of the list, while words at the top of the list have a small change in their rank. Other choices may change the rank of the words in all parts of the list significantly. Table 2 presents another retrieved list of words according to this Combined Measure. Now, words like slaves, wax, hybrids, and instincts are placed in the top. In this new ranking list, the word, hybrid, changes its place from 321 to 48, the word, rarely also moves from 2203 rank to 1011.

**Table 2:** List of the twenty top-ranked words according to Combined Measure from the book *The Origin of Species*. These words are important according to the subject of the book. The word, *f* is related to some classification of species such as f8, f10, f14, … and some proper names. *f* is kept because non-alphabetical characters are removed in our method.

Words	Frequency	Fractality	Combined Measure
slaves	34	17.42	26.68
wax	42	15.65	25.40
hybrids	135	10.89	23.20
instincts	87	11.85	23.00
sterility	100	11.27	22.53
cuckoo	32	14.89	22.40
illegitimate	21	16.52	21.85
floated	18	15.98	20.07
instinct	63	10.62	19.11
masters	17	15.52	19.10
lamellae	20	14.67	19.09
pedicellariae	15	16.03	18.85
cell	28	12.71	18.39
nest	55	10.23	17.80
f	46	10.62	17.66
pupae	13	15.72	17.51
cells	58	9.84	17.36
fertility	80	9.08	17.27
spheres	19	13.46	17.22
clover	15	14.55	17.11

In addition to the degree of fractality, there exist several methods that assign an importance value to any word in a given text. We can list the words in descending order of their importance. In this list the words that are placed in the top ranks are assumed to be keywords. By choosing a threshold value we can identify the list of keywords. In the following section we evaluate our proposed method for the keyword detection task.

### Evaluation of Our Method

The best way to evaluate the efficiency of our approach to keyword detection is comparing its results with other methods. We use two metrics in this comparison: precision and recall. These tell us to what extent the retrieved list of keywords conforms to the manually selected list as described in the previous section. In this work, we would like to compare our method with two efficient methods in keyword extraction, the *C Value* [14] and *Entropy* [17]. These methods are selected according to our experience. We found that C Value has the maximum amount of recall compared with other methods and entropy has maximum amount of precision compared with others [18, 23] (these methods are reviewed in further detail in the appendix). To do the assessment we use the glossary written by W. S. Dallas [24]. Note that the choice of glossary has the potential to considerably alter the result of comparisons.

Two points are relevant before proceeding to the comparison. First, the glossary of the book contains not only words, but also some phrases. To deal with multi-word keywords of the glossary we separate them into single words. For example we convert the phrase ganoid fishes to two separate words ganoid and fishes in the glossary. Second, in any method, a value is assigned to each vocabulary word, then we can sort the words from the highest value to the lowest. We give rank 1 to the first word in the sorted list, the second word takes rank 2 and so on. Unlike in Zipfian ranking, this ranking process allows for rank ties; in other words, if some words have the same assigned value, they should have the same rank. As an example, in Table 3 the words forward and months have equal values. In this case we assign them equal rank (2128) and the next word in the list will have rank 2130. There are two approaches for calculating recall and precision.

**Table 3:** List of ten words and their ranks from the book *The Origin of Species*. Words with equal Combined Measures take equal ranks.

Words	Combined Measure	rank
forward	3.31199	2128
months	3.31199	2128
saved	3.31115	2130
treat	3.31115	2130
observers	3.30809	2132
gone	3.30749	2133
inferiority	3.30647	2134
agree	3.30564	2135
icebergs	3.30447	2136
laying	3.30447	2136
really	3.30164	2138

**Fig 9:**
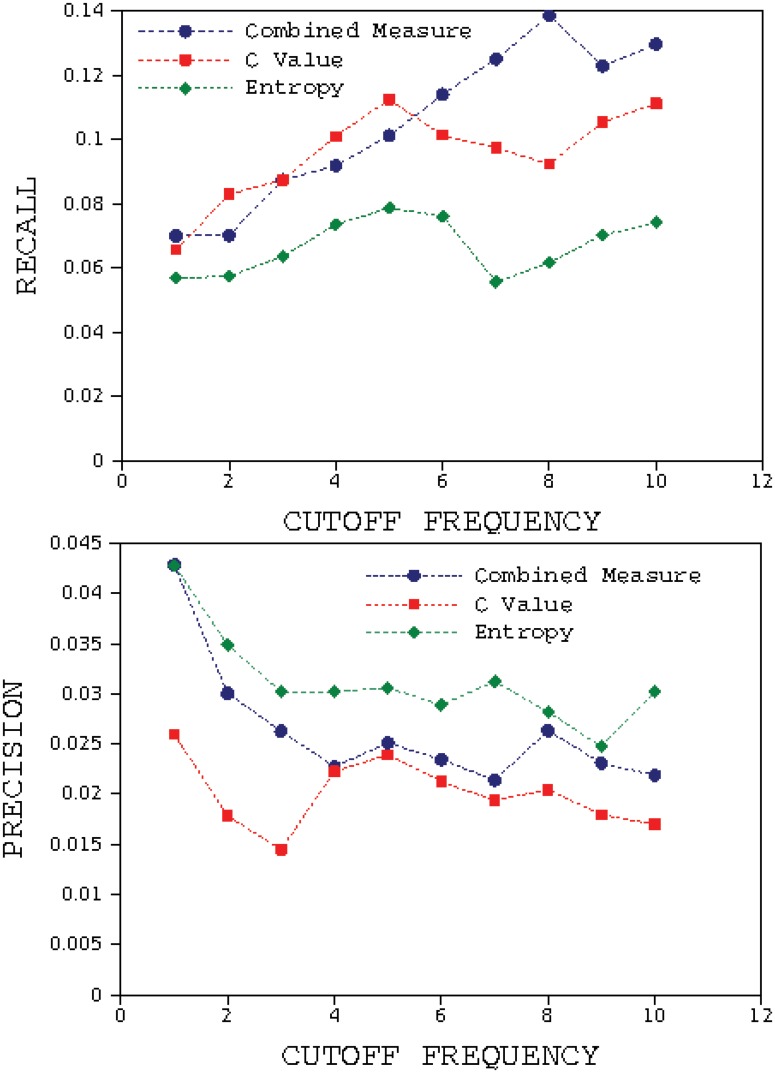
Results of calculating Recall and Precision with Herrera and Purri approach for the book *The Origin of Species* for 10 cut-off frequencies. The fractal method has the highest value of Recall in all frequencies and higher value of Precision than C Value method.

In Herrera and Puri approach [17], they do not indicate any threshold. After ranking words according to an importance index, the last word of the glossary in the ranked list is found. Then, the number of words from the ranked list which include all the glossary words are selected as keywords. In this approach, they introduce a cut-off frequency; they keep only the words with frequencies greater or equal to the cut-off frequency both in ranked list and in the glossary and omit all other words with lower frequencies. For example cut-off frequency equal to 2 means only words with frequencies more than 1 are kept and other words are omitted. The number of words from ranked list and from the glossary for various choices of cut-off frequency are written in Table 4. In Fig 9, the recall and precision are plotted against the cut-off frequency. According to Fig 9, recall for Combined Measure is higher than other methods for cut-off frequencies greater than 5. This means that the proposed fractal method is superior to the others as a method for keyword extraction. The precision of Combined Measure is higher than C Value for all cut-off frequencies.

**Table 4:** Number of vocabulary words and number of glossary words for various cut-off frequencies. *N*_*v*_ and *N*_*g*_ are the number of vocabulary words from the book and number of glossary words for each cut-off frequency, respectively.

	Cut-off Frequency									
	1	2	3	4	5	6	7	8	9	10
*N*_*v*_	8842	5351	4092	3428	2957	2624	2352	2141	1968	1855
*N*_*g*_	229	157	126	109	89	79	72	65	57	54

**Fig 10:**
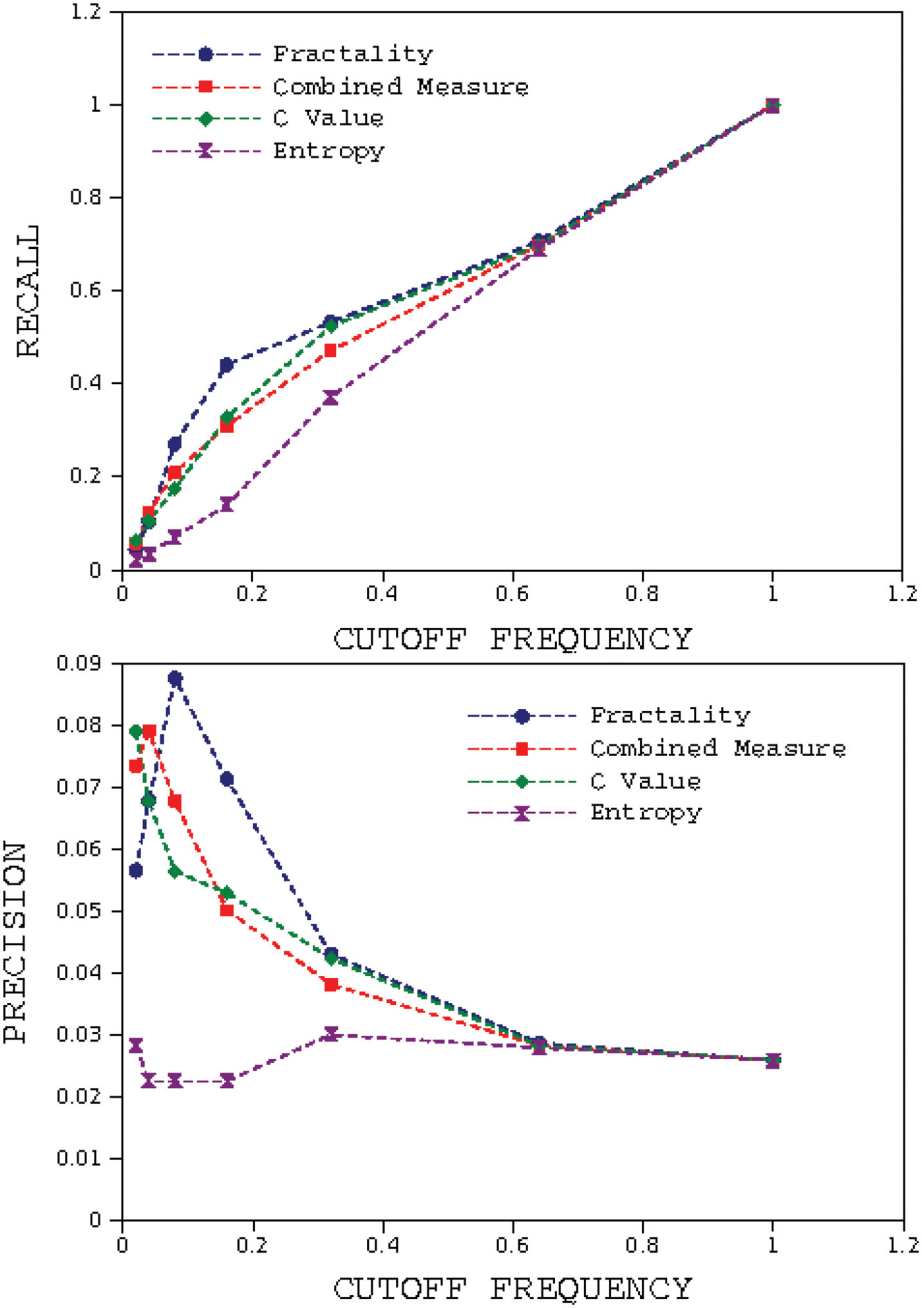
Results of calculating Recall and Precision with Mehri and Darooneh approach for the book *The Origin of Species*. The fractal method has the highest value of Recall and precision in all vocabulary fractions.

If we rank the words according to their fractality we will find a power law relationship between the fractality of a word and its rank. Therefore, it is rational to choose the words with rank lesser than a specific value as the retrieved keywords list instead of using the fractality threshold. In Mehri and Darooneh approach [18], after ordering words due to their fractality, a percentage of words from the top of the ranked list are selected as keywords. In the first step, the top 2 percent of the ranked list are selected as keywords (the first 2 percent of 8842). In the next step, the top 4 percent of the list are selected as keywords, and so on. Also, in this approach all of the glossary words are selected as relevant keywords in all steps. In Fig 10, the recall and precision are plotted using Mehri and Darooneh approach. According to this figure recall for fractality for our method is higher than other methods for all retrieved list fractions. The precision of fractality for our method is higher than others for retrieved list fractions of more than 4 percent.

The validity of our method also extends to other books. *The First Three Minutes* by Steven Weinberg [25] and *A Brief History of Time* by Stephen Hawking [26]. The value of recall for our method is higher than for C Value and entropy. The precision value obtained is higher than other methods for cut-off frequencies of more than 9 for Weinberg’s book, and more than 8 in the case of Hawking’s book.

## Conclusion

The pattern of occurrences of a word in a text can be considered as a fractal object with dimension between 1 and 0. We found that words related to the subject of the text have non-uniform spatial distributions and their dimensions are considerably less than one. In contrast, the irrelevant words are distributed uniformly with a dimension close to one. We introduced the concept of degree of fractality which measures the difference between distribution pattern of a word in the original text and randomly shuffled version. While in the shuffled texts all of the words are uniformly distributed across the text, the original text exhibits clustering of important words in particular. We used the degree of fractality in combination with a function of frequency for ranking words in *The Origin of Species* by Charles Darwin. The top words in the ranked list of the words was selected as the retrieved keywords of the text. The retrieved list of keywords was checked against the glossary of the book. For this checking we used two metrics: precision and recall, which are defined in the context of the binary classification analysis. Compared with two other representative methods in this area, the Entropy and C Value, our approach is more effective as a method for automatic keyword extraction.

Future work should aim to examine the effectiveness of our method in keyword detection for smaller texts. This method could also be applied to key-phrase extraction. Finally, the general framework behind our method could be extended to explore the hidden secrets of genome, for instance by developing a way for data mining non-coding DNA.

## Appendix. Description of related methods of word ranking

### A C Value

The C Value method is based on noticing distribution of the words in a text and word clustering [14]. To quantify the clustering of a word the parameter *σ* (the standard deviation of the normalized distance between consecutive occurrence of a word) is defined by

$$\begin{array}{c} \sigma =\sqrt{<s^{2}>-{<s>}^{2}}, \end{array}$$ (9)

Where *s* is the normalized distance between consecutive occurrences, *s* = *d*/ < *d* >, and < *d* > is the average distance between occurrences. *σ* can be normalized with respect to standard deviation of the distance between consecutive occurrences of words in a random text, which has a geometrical spatial distribution of word types, $\sigma_{geo}=\sqrt{1-P}$. Where *p* = *M*/*N* is the probability of occurrence of a word type with frequency equal to M in a text with total N words,

$$\begin{array}{c} \sigma_{nor}=\frac{\sigma }{\sigma_{geo}}, \end{array}$$ (10)

$$\begin{array}{c} C\left(\sigma_{nor},M\right)=\frac{\sigma_{nor}-<\sigma_{nor}\left(M\right)>}{sd\left(\sigma_{nor}\right)\left(M\right)}, \end{array}$$ (11)

Where $<\sigma_{nor}>=\frac{2M-1}{2M+1}$ and $sd(\sigma_{nor})=\frac{1}{\sqrt{M}(1+2.8M^{-0.865})}$ are the mean value of the normalized standard deviation and standard deviation of the distribution of *σ*_*nor*_ in a random text, respectively. *C* = 0 means the word is distributed randomly in a text and *C* > 0 means the word forms cluster.

### B Entropy

Entropy is another parameter used to rank the words of a text [17]. For this purpose a text with N words is devided into P parts. The *i*th part contains *N*_*i*_ words which $\sum_{i=1}^{P}N_{i}=N$. So the ralative frequency of occurrence of the word type *ω* in the part *i* is $f_{i}(\omega )=\frac{M_{i}(\omega )}{M(\omega )}$, where *M*_*i*_(*ω*) and *M*(*ω*) are the frequency of word type *ω* in the ith part and in the whole text, respectively, where $\sum_{i=1}^{P}M_{i}=M$. With this explanation the probability measure over the partitions can be defined as

$$\begin{array}{c} p_{i}\left(\omega \right)=\frac{f_{i}\left(\omega \right)}{\sum_{j=1}^{P}f_{j}\left(\omega \right)}. \end{array}$$ (12)

The following relation is the Shannon’s information entropy for a discrete distribution *p*_*i*_(*ω*)

$$\begin{array}{c} S\left(\omega \right)=\frac{-1}{Ln(P)}\sum_{i=1}^{P}p_{i}\left(\omega \right)Ln\left(p_{i}\left(\omega \right)\right). \end{array}$$ (13)

There is a problem with this relation; it is zero for words with frequency equal to 1. To take into account the effect of frequency, the following relation seems to be a better choice

$$\begin{array}{c} E_{nor}\left(\omega \right)=\frac{M(\omega )[1-S(\omega )]}{E_{ran}\left(\omega \right)} \end{array}$$ (14)

where $E_{ran}(\omega )=\frac{P-1}{2ln(P)}$ is the entropy of the word type *ω* in a random text.
